# Arginase Isoform Expression in Chronic Rhinosinusitis

**DOI:** 10.3390/jcm8111809

**Published:** 2019-11-01

**Authors:** Diana Vlad, Silviu Albu

**Affiliations:** 1 Second Department of Otolaryngology, University of Medicine and Pharmacy Cluj-Napoca, Cluj-Napoca 400489, Romania; silviualbu63@gmail.com; 2 Department of ENT, University of Medicine and Pharmacy Iuliu Hatieganu from Cluj-Napoca, Cluj Napoca 400006, Romania

**Keywords:** arginase, arginase isoform, chronic rhinosinusitis, nitric oxide

## Abstract

Nitric oxide (NO) has emerged as an important regulator of upper airway inflammation, mainly as part of the local naso-sinusal defense mechanisms. Increased arginase activity can reduce NO levels by decreasing the availability of its precursor, L-arginine. Chronic rhinosinusitis (CRS) has been associated with low levels of nasal nitric oxide (nNO). Thus, the present study investigates the activity of arginase I (ARG1) and II (ARG2) in CRS and its possible involvement in the pathogenesis of this disease. Under endoscopic view, tissue samples of pathologic (*n* = 36) and normal (*n* = 29) rhinosinusal mucosa were collected. Arginase I and II mRNA levels were measured using real-time PCR. Our results showed low arginase I activity in all samples. The levels of ARG2 were significantly higher in patients with chronic rhinosinusitis compared to the control group (fold regulation (FR) 2.22 ± 0.42 vs. 1.31 ± 0.21, *p* = 0.016). Increased ARG2 expression was found in patients with CRS without nasal polyposis (FR 3.14 ± 1.16 vs. 1.31 ± 0.21, *p* = 0.0175), in non-allergic CRS (FR 2.55 ± 0.52 vs. 1.31 ± 0.21, *p* = 0.005), and non-asthmatic CRS (FR 2.42 ± 0.57 vs. 1.31 ± 0.21, *p* = 0.028). These findings suggest that the upregulation of ARG2 may play a role in the pathology of a distinctive phenotype of CRS.

## 1. Introduction

Despite being a well-known, prevalent medical condition with significant socioeconomic implications [1], the pathology of chronic rhinosinusitis (CRS) currently remains a matter of great debate. This chronic inflammatory process is considered to be multifactorial [2]. Confounding factors that are involved in this process include: local dysfunctional pathways of inflammatory regulation and resolution, microbial (biofilms, osteitis) and fungi colonization, superantigens, allergy, and other abnormal adaptive immunologic responses and metabolic abnormalities such as aspirin sensitivity. 

**Figure 1:**
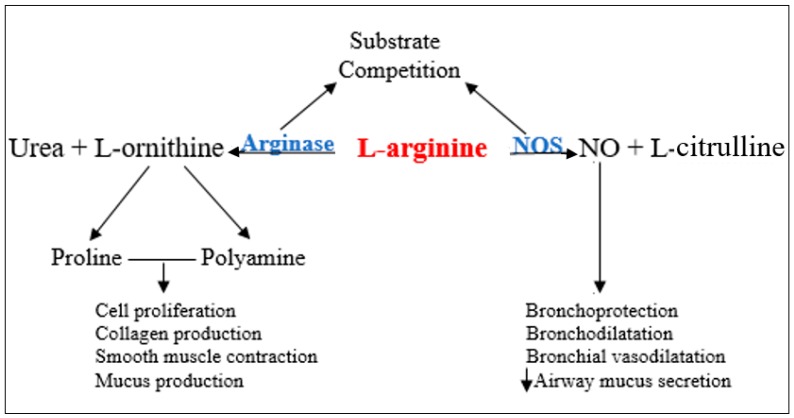
Nitric oxide (NO) pathway. NOS—nitric oxide synthase.

Lately, nitric oxide (NO) has gained a central role in local chronic inflammatory reactions. The paranasal sinuses are considered a major source of NO that is held to ensure a protective role for the upper (antibacterial, antiviral, antifungal, increases mucociliary clearance and modulates inflammatory response) [3,4,5] and lower airways [5]. Nasal NO levels have been reported to be low in patients with chronic [4,6] or acute rhinosinusitis [7] and primary ciliary dyskinesia [8], but high in allergic rhinitis [9]. The endogenous biosynthesis of NO is the result of L-arginine oxidation, a reaction catalyzed by inducible nitric oxide synthase (NOS) (Figure 1). L-arginine is an amino acid also used by arginase (ARG) in the urea cycle to form urea and ornithine [10], thus the two enzymes are competing for the same substrate. Arginase exists in two isoforms: arginase-1 (ARG1) and arginase-2 (ARG2). There are several processes that control the expression of ARG1 and ARG2 genes, and it appears that these processes are involved in the pathogenesis of numerous airway diseases via L-arginine–nitric oxide pathway modulation [11,12,13,14,15]. 

Currently, CRS is classified according to the 2012 European Position Paper on Rhinosinusitis (EPOS) which defines three subtypes of sinusitis: (1) CRS with nasal polyps (CRSwNP), (2) CRS without nasal polyps (CRSsNP), and (3) allergic fungal sinusitis (AFS) [16]. Recently, based on the clinical, histological, and biomolecular features, Han has elaborated a more relevant subclassification of this disease into seven distinct groups designed for individualized management and research purposes: aspirin-exacerbated respiratory disease (AERD), asthmatic sinusitis with and without allergy, non-asthmatic sinusitis with and without allergy, allergic fungal sinusitis, and cystic fibrosis (CF) [17].

In the present case-control study, we assessed the sinus mucosa expression levels of ARG1/2 in patients with CRS, with an emphasis on the differences found between the different subtypes of chronic rhinosinusitis according to Han’s classification, its impact on disease pathology and further clinical opportunities. 

## 2. Materials and Methods

### 2.1. Protocol

Experimental data were prospectively collected in the period of August–December 2016 in the ENT Department of the CF Clinical Hospital Cluj-Napoca. The experimental group included patients who met the following inclusion criteria: patients diagnosed with chronic rhinosinusitis based on disease history, imaging by cranial CT, and nasal endoscopy, patients in whom optimally administered drug therapy proved to be ineffective. The control group included patients undergoing endoscopic skull base procedures, without associated sinus diseases. Patients aged less than 18 years, immunocompromised patients, patients with ciliary dyskinesia and patients receiving antibiotics or corticosteroids 4 weeks before admission were excluded from the study.

All patients enrolled in the study were informed about the evaluation and therapeutic procedures applied, and they gave their written informed consent for participation. The present study obtained the approval of the Ethics Committee Iuliu Hatienganu Cluj-Napoca.

CT images were classified based on the Lund–Mackay scoring system [18]. 

The diagnosis of bronchial asthma in the studied patients was confirmed by a pulmonologist based on GINA criteria [19]. Allergy tests were performed on all patients by an allergist or dermatologist, and the diagnosis of allergy was established based on existing clinical guidelines [20,21]. 

### 2.2. Classification

The case group was divided, based on clinical characteristics, into 4 distinct subgroups representing different phenotypic and biomolecular subtypes of chronic rhinosinusitis, consistent with Han’s subclassification of CRS: (1) non-asthmatic sinusitis without allergy, (2) non-asthmatic sinusitis with allergy, (3) asthmatic sinusitis without allergy, (4) asthmatic sinusitis with allergy [17]. For the purpose of this study, patients with aspirin triad, fungal CRS, or cystic fibrosis were not included in the investigation.

### 2.3. Sample Collection

Under endoscopic control, small fragments of respiratory mucosa affected by inflammation were collected from the ethmoid sinus of each patient in the experimental group. In the control group, biopsies were also taken from the ethmoid sinus.

The tissue samples were placed in RNAlater (Thermo Fisher Scientific, Waltham, MA, USA) solution in order to reduce RNase enzyme activity with a role in RNA degradation. Then, the biological samples were washed with phosphate buffer solution (PBS 1×), transferred to Nalgene tubes, and stored in liquid nitrogen until their subsequent use.

### 2.4. Genetic Assessment

From the tissue specimens, total RNA was extracted by the classic method (phenol–chloroform) according to standardized protocols. The extracted RNA was purified in specific extraction columns according to the method described by Qiagen (RNeasy Mini kit Qiagen, Venlo, The Netherlands). The amount of RNA was assessed with a Nanodrop-ND-1000 spectrophotometer (Thermo Fisher Scientific, Waltham, MA, USA) based on two successive readings. Total RNA was qualitatively assessed with a 2100 Bioanalyzer (Agilent Technologies, Santa Clara, CA, USA) using the Lab-on-a-chip technology. All samples with a minimum concentration of 100 ng/μL RNA and a RIN (RNA integrity number) value ˃ 8 were included in subsequent analyses for gene expression evaluation.

For gene expression analysis, the Roche LightCycler 480 (Hoffmann-La Roche AG, Basel, Switzerland) was used. For the genes of interest, based on Universal ProbeLibrary Assay Design Center studies in silico, a specific set of primers and UPL (Universal Probe Library) probes was established, so as to meet the best PCR amplification conditions. The expression levels of the genes of interest were normalized to the housekeeping 18s gene. 

The analysis of gene expression levels was conducted using the ΔΔCt method, with the expression levels obtained in the control group (healthy subjects) as a reference. The fold regulation (FR) value for each group was calculated relative to the control group, after normalization to the housekeeping gene 18s.

### 2.5. Statistical Analysis

The data were collected in an Excel database. For dichotomous data, the chi-square test or Fisher’s test was used, and for the analysis of ordinal data, the two-way Kruskal–Wallis test, followed by the Mann–Whitney U test, was employed. In the case of abnormal distributions, the difference in gene expression between the studied groups was evaluated by the non-parametric Kruskal–Wallis test, followed by the Dunn test for multiple comparisons. Differences were considered statistically significant at a *p* value < 0.05.

## 3. Results

In this study, 65 samples from 36 patients diagnosed with chronic rhinosinusitis and 29 control patients with septum deviation were analyzed. The general characteristics of the patients are described in Table 1.

**Figure 2:**
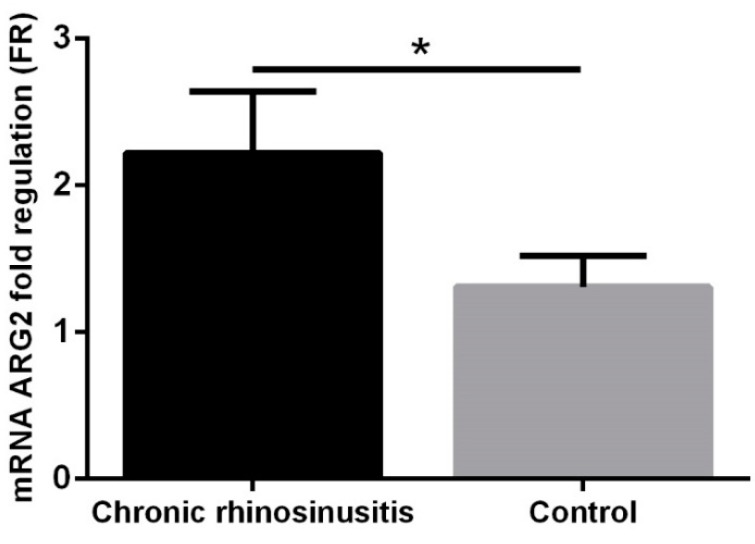
Arginase-2 (ARG2) expression in the group with chronic rhinosinusitis and in the control group. * *p* < 0.05.

ARG1 expression levels were globally low in the group of patients with chronic rhinosinusitis and in the control group, which is why these were not considered for statistical analysis. In patients with chronic rhinosinusitis, significantly higher ARG2 values were observed compared to the control group (FR 2.22 ± 0.42 vs. 1.31 ± 0.21, *p* = 0.016, Figure 2). 

**Figure 3:**
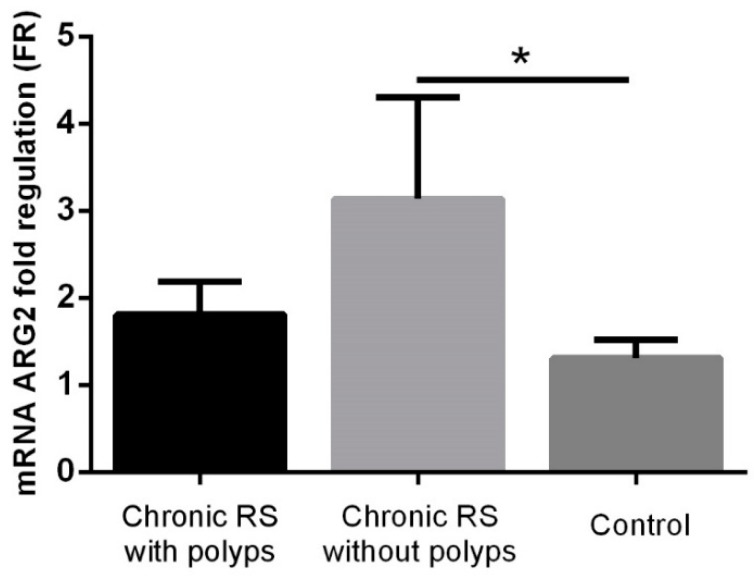
ARG2 expression in the group with chronic rhinosinusitis (RS) (with or without polyps) and in the control group. * *p* < 0.05.

Increased ARG2 levels were found in patients with chronic rhinosinusitis without polyposis compared to the control group (FR 3.14 ± 1.16 vs. 1.31 ± 0.21, *p* = 0.0175), while in patients with chronic rhinosinusitis and polyposis, although high ARG2 levels were detected (FR 1.81 ± 0.38), these were not significantly increased compared to the control group (Figure 3). 

**Figure 4:**
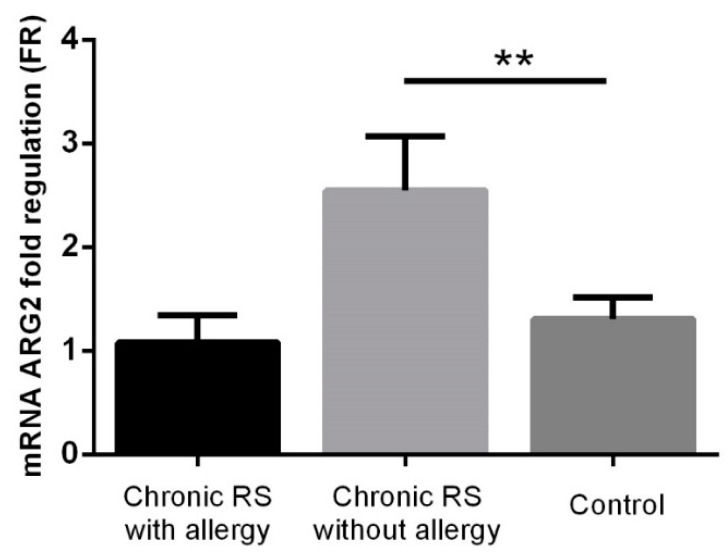
ARG2 expression in the group with chronic rhinosinusitis (with or without allergy) and in the control group. ** *p* < 0.005.

Significantly increased ARG2 values were identified in non-allergic patients with chronic rhinosinusitis compared to the control group (FR 2.55 ± 0.52 vs. 1.31 ± 0.21, *p* = 0.005), while in the case of allergic patients with chronic rhinosinusitis, ARG2 values (FR 1.08 ± 0.26) were lower than in the control group, but without reaching the statistical significance threshold (Figure 4). 

**Figure 5:**
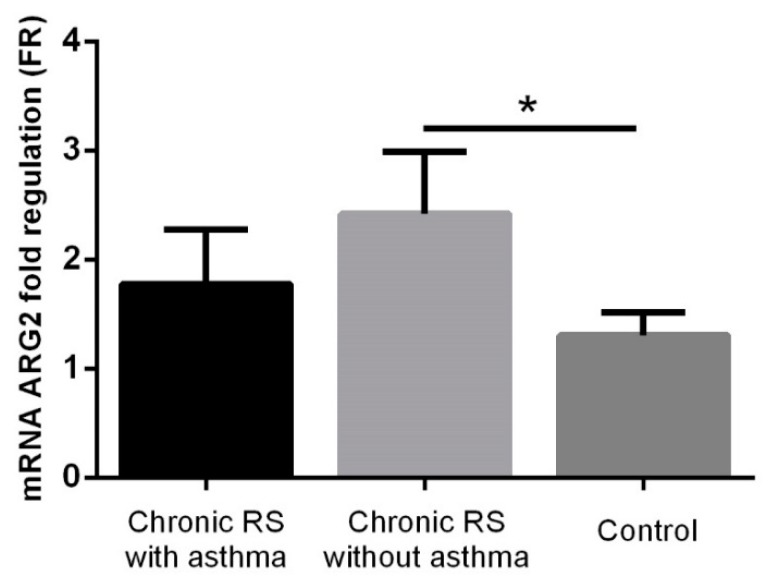
ARG2 expression in the group with chronic rhinosinusitis (with or without asthma) and in the control group. * *p* < 0.005.

ARG2 values were statistically increased in non-asthmatic patients with chronic rhinosinusitis compared to the control group (FR 2.42 ± 0.57 vs. 1.31 ± 0.21, *p* = 0.028), while in asthmatic patients with chronic rhinosinusitis, although high ARG2 levels were observed (FR 1.78 ± 0.5), these were not significantly increased compared to the control group (Figure 5).

**Figure 6:**
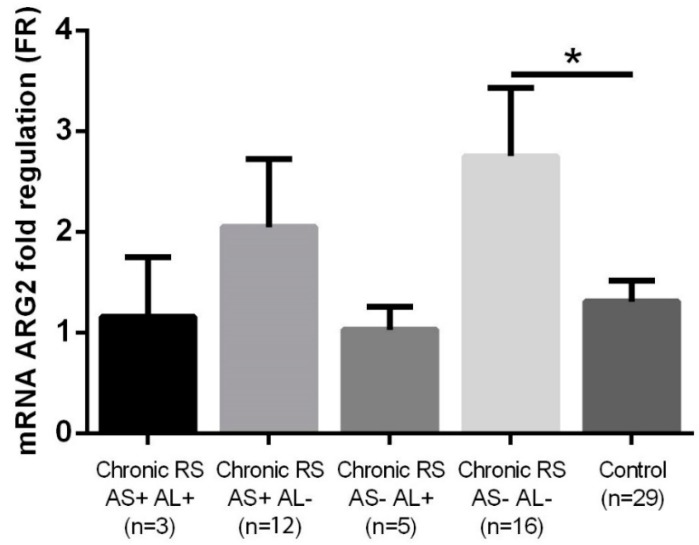
ARG2 expression in the group with chronic rhinosinusitis (with or without asthma/allergy) and in the control group. * *p* < 0.005, AS+ asthmatic, AS− non-asthmatic, AL+ allergic, AL− non-allergic.

Statistically significantly higher ARG2 levels were also identified in non-allergic, non-asthmatic patients with chronic rhinosinusitis compared to the control group (FR 2.75 ± 0.68 vs. 1.31 ± 0.21, *p* = 0.025). In the other subgroups of patients with chronic rhinosinusitis (allergic asthmatic, non-allergic asthmatic, allergic non-asthmatic), no statistically significantly different ARG2 values were found (FR 1.15 ± 0.59, 2.05 ± 0.67, 1.03 ± 0.23) compared to the control group (Figure 6). 

**Figure 7:**
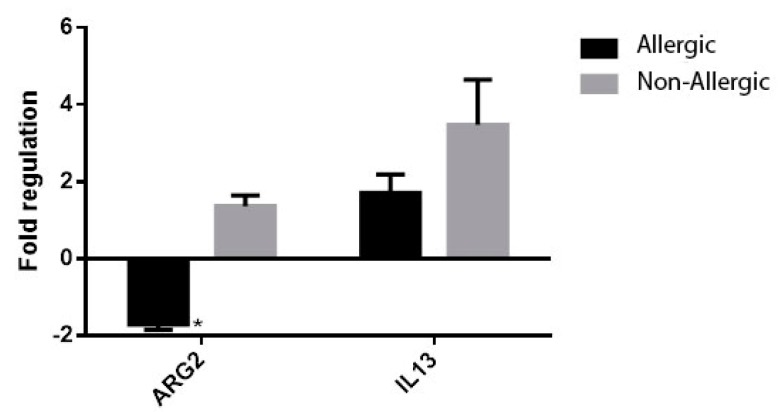
ARG2 and interleukine-13 (IL-13) expression in patients with chronic rhinosinusitis (CRS): allergic vs. non-allergic, * *p* = 0.003.

**Figure 8:**
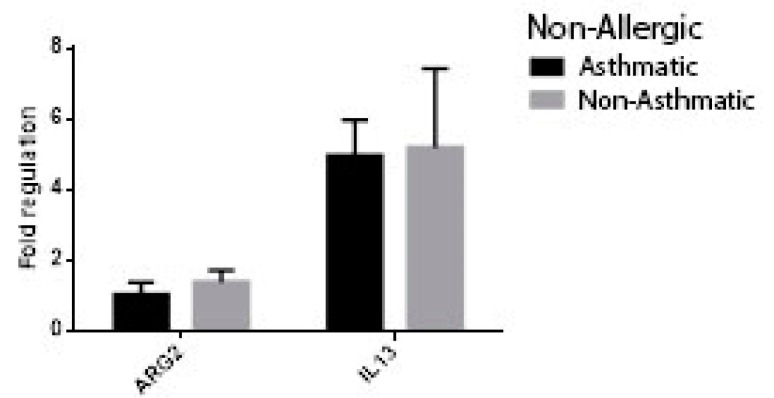
ARG2 and IL-13 expression in patients with non-allergic CRS: asthmatic vs. non-asthmatic, *p* = 0.02.

**Figure 9:**
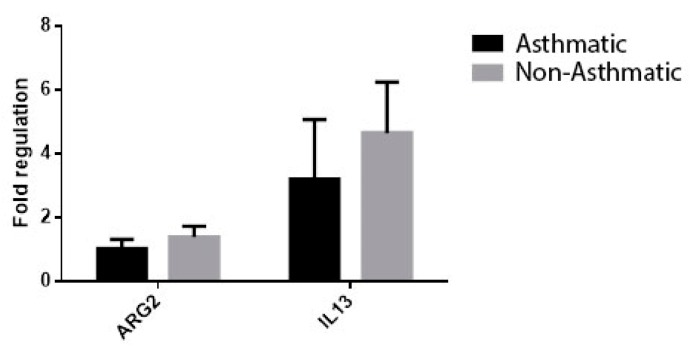
ARG2 and IL-13 expression in patients with CRS: asthmatic vs. non-asthmatic. *p* = 0.23.

**Figure 10:**
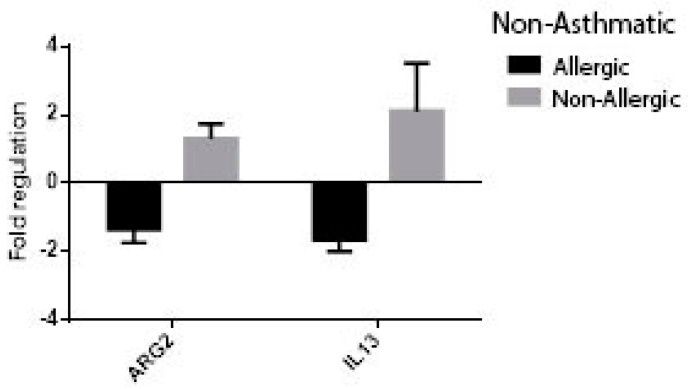
ARG2 and IL-13 expression in patients with CRS and no asthma: allergic vs. non-allergic. *p* = 0.27.

At univariate analysis of patients with chronic rhinosinusitis using the following comparison groups: allergic vs. non-allergic, asthmatic vs. non-asthmatic, non-asthmatic allergic vs. non-asthmatic non-allergic, non-allergic asthmatic vs. non-allergic-non-asthmatic, the expression of ARG2 was significantly higher in patients with non-allergic CRS (FR −1.71 ± 0.14 vs. 1.37 ± 0.28, *p* = 0.003) (Figure 7) and non-asthmatic non-allergic patients (FR 1.03 ± 0.34 vs. 1.38 ± 0.34, *p* = 0.020, Figure 8). Though higher values of ARG2 were likewise found in the subgroups of patients with CRS and no asthma (Figure 9, Figure 10), the results were not statistically significant. The expression of interleukine-13 (IL-13), as a marker of inflammation in chronic sinusitis, was also determined and included in the analysis; however, we found no correlation in the growth pattern between the expression of ARG2 and IL-13.

## 4. Discussion 

Nitric oxide is an important airway mediator, and its role in chronic rhinosinusitis represents a subject of high interest. It is produced mainly by the nasal and bronchial epithelial and inflammatory cells under the control of inducible NO synthase (iNOS or NOS2) [22]. NO is part of the first-line host defense mechanisms; its high concentration in the paranasal sinuses can reach up to 23.000 ppb [10], ensuring an antibacterial, antiviral, and antifungal effect. NO increases mucociliary clearance by upregulating ciliary motility; an association between low levels of NO and impaired mucociliary transport has been established [23,24]. Importantly, nitric oxide can display a pro- or anti-inflammatory effect conditioned by the type and phase of airway inflammation, its local concentration, and the individual response [25]. In the respiratory tract, nitric oxide has been shown to be involved in both T helper type 1 (Th1) and Th2 immunologic response [5,26,27]. Th1 lymphocytes secrete interleukin (IL)-2 and interferon-gamma (INF-γ); they are associated with non-eosinophilic inflammatory diseases and intense phagocytic activity [28,29]. Th2 are closely involved in allergy; their immune response involves the production of IL-4, IL-5, and IL-13, which lead to the recruitment and activation of mast cells, basophils and eosinophils, and goblet cell hyperplasia in airway mucosa [29,30]. Low amounts of NO have been shown to stimulate Th1 differentiation and INF-γ release, while NO in higher concentrations can induce Th2 proliferation while promoting Th1 apoptosis [26,31]. Clearly, NO is a key effector molecule in the Th1/Th2 dichotomy that regulates the evolution of many important diseases. 

In patients with CRS, NO levels are reduced. Its decrease is consistent with the severity of the disease, and a concentration increase was noted after therapeutic measures were applied [4,10,32]. The low levels of exhaled and nasal NO found in patients with CRS have been attributed to various mechanisms: (1) sinus ostium obstruction and increased mucosal absorption, injury to the NO-producing sinus mucosa by increased production of cytotoxic agents in chronic inflammation, or (3) decreased iNOS expression caused by certain cytokines, such as IL-4, IL-6, and TGF-β, found in the sinus mucosa of patients with CRS [32,33,34]. A recent pertinent principle is that increased arginase activity can lead to a limitation of nitric oxide synthesis through the privation of l-arginine, the precursor substrate for NOS, as shown in Figure 1. The balance between ARG and NOS activity is responsible for the resulting nasal NO levels and its effect on local homeostasis.

Arginase can be found in two isoforms: arginase I (cytosolic) and arginase II (mitochondrial) [35]. We investigated the expression of ARG1/ARG2 in sinusal mucosa of patients with CRS. Only one other study, conducted by Taruya et al. [4], has also analyzed arginase in chronic sinusitis. We observed that arginase 1 had a low expression in both the group of CRS cases and in the control group. Although there is high evidence that increased pulmonary arginase 1 activity plays an important role in perpetuating and/or potentiating eosinophilic inflammatory lung disease, assumed to be via the decrease of NO production [36,37,38], in our study nasal ARG 1 had a low expression in all types of CRS. It has not been proven to be a key factor in CRS pathogenesis as it has been in the lower airways. Taruya et al. found similar results in their study on 45 cases [4]. Arginase 1 impact in asthma might be associated to a greater extent with other, non-NO related, reactions conditioned by this enzyme. 

Regarding ARG2, a significantly higher expression was found in the group of patients with chronic rhinosinusitis compared to the control group, sustaining its repercussion on sinusitis pathology. Subgroup analysis demonstrated that significantly higher arginase 2 values were reached in the subgroup of patients without polyps, consistent with Taruya’s outcomes [4], which showed a correlation between high ARG2 expression and lower exhaled NO values. An increased ARG2 activity reduces NO synthesis, thus stimulating Th1 differentiation, non-eosinophilic inflammation, and macrophage activation (which are ultimately compromised in NO release due to absent substrate). ARG2 expression was lower in CRSwNP than CRSsNP, but higher than that found in the control group. Both varieties of CRS display a cellular infiltrate of neutrophils, macrophages, and lymphocytes, as well as Th1 proinflammatory cytokines [39]. CRSwNP is distinguished by an eosinophilic inflammatory infiltrate and Th2-type cytokine predominance [40,41]. As mentioned above, superior NO values stimulate Th2 proliferation, IgE production and eosinophil recruitment. It is very plausible that moderate levels of ARG2 partially inhibit NO production so that it can no longer assure a protective role but allow NO accumulation in concentrations that are sufficient to promote Th2 differentiation. Various studies indicate that the low nasal nitric oxide (nNO) concentration in CRSwNP is the consequence of osteomeatal obstruction [42]. The possible increase of NO in an obstructed sinus leads to Th2 overstimulation and contributes to a cascade of local reactions that ultimately perpetuate eosinophilic inflammation. In this regard, Lee et al. [43] recently proved that nNO values of CRSwNP patients significantly increased after endoscopic sinus surgery (ESS), while remaining constant in CRSsNP. Currently, nNO is considered a marker of sinus mucosal health following ESS in CRSwNP. Nonetheless, our findings are more supportive of the involvement of ARG2 in non-Th2 mediated inflammation in CRS pathology.

However, there is growing evidence that regulatory T cells (Tregs) are important in controlling and directing mucosal immune responses, and impairment of Tregs is hypothesized to further contribute to CRSwNP pathogenesis [39]. In vitro and in vivo studies have observed that NO, through the generation of NO-Tregs, plays a central role in regulating the strength of chronic inflammatory responses associated with autoimmune and allergic diseases. Ongoing research aims to shed more light on to this topic.

The highest levels of arginase 2 were observed in the non-asthmatic sinusitis without allergy group (NASsA), according to Han’s subclassification. Characteristically, patients with NASsA have an extrinsic non-eosinophilic inflammatory reaction caused by prolonged sinus infection. They have purulent secretion on nasal endoscopy and low CT scores. The immune response seems to be Th1 mediated, involving elevated levels of IFN-γ and also high IL-6 and IL-8, comparable to non-eosinophilic sinusitis [17]. 

Th1 cells produce IFN-γ, which stimulate macrophages to synthetize high levels of NO, with an antimicrobial effect, and meanwhile NO selectively inhibits Th1 response and production of IFN-γ [31], preventing an exacerbated local host reaction. Disturbance of this regulatory feedback pathway by ARG2 interference would lead to the perpetuation of an inefficient inflammatory response to local infection. NO also inhibits Th1 proliferation by blocking macrophage-derived IL-12 release [44]. Furthermore the immune-cell production of various cytokines, like IL-1, IL-6, IL-8, IFN-γ, and TNF-α, can be inhibited by iNOS-derived NO [27], thus emphasizing the connection between high arginase 2 activity, low NO levels, and high IFN-γ, IL-6, and IL-8 demonstrated in patients with non-asthmatic sinusitis without allergy. Additionally, interleukin-8 (IL-8) was observed to be able to directly upregulate the expression and exocytosis of ARG1 and ARG2 in various cell types, so it could likewise manifest on sinusal epithelial or local inflammatory cells, perpetuating a cyclic inflammatory cycle [45,46]. 

When correlating arginase 2 values in patients without polyps with the Lund–Mackay CT score, we observed that patients with more severe sinus disease had greater ARG2 levels. This is consistent with other prior studies that showed a negative correlation between nNO and disease severity on CT [32,47]. This supports our findings that arginase/NOS imbalance can be responsible for an abnormal inflammatory reaction due to a lack of pathway regulation and/or the local perpetuation of microorganisms owing to reduced NO synthesis and mucociliary dysfunction. ARG2/resulting metabolites could be implicated in additional proinflammatory sinusal responses. From a clinical standpoint, this would assist the identification of patients in need of a more adapted treatment that could improve long-term outcomes.

The exact role of arginase and arginase/NO balance in the pathophysiology of the upper respiratory tract remain a matter for further research. Data accumulated so far suggest its central role in lower airway hyperreactivity, inflammation, and remodeling through a decrease in nitric oxide levels, which demonstrate an anti-inflammatory and bronchodilatatory role, as well as through an accumulation of L-ornithine, involved in cell proliferation and collagen synthesis [37]. Nonetheless, the role of ARG in the lower airways could be attributed, to a larger extent, to its involvement in other inflammatory pathways. Other possible mechanisms related to arginase modulation of local respiratory immune responses remain to be clarified.

Our study supports the need for a more comprehensive classification of patients with CRS due to its clinical heterogeneity and complex pathogenesis characterized by a great diversity of immunological mechanisms and possible etiological factors. Han’s subclassification is a pertinent undertaking based on different phenotype characteristics of patient with CRS and the differences in biomarker expression with intracellular staining of cytokines that deserves further acknowledgement. Although our study was limited by the small number of patients distributed to each subtype of CRS, the implication of these results warrants significant consideration. This may have future research implications towards a better understanding of this complex disease and the development of novel therapeutic strategies.

By defining more accurately distinct pathological subclasses of patients with CRS, individualized management and improved targeted treatment can be developed for each category. Treatment of NASsA implies long-term corticosteroid and macrolide therapy [17], as well as endoscopic sinus surgery in failed cases. Nonetheless, there still remain a significant number of patients who continue to suffer from persistent chronic sinus inflammation despite optimal treatment. Related to our study, research on animal models with asthmatic inflammation involving the administration of arginase inhibitors or L-arginine supplementation have shown a reduction in the severity of airway inflammation and the development of airway hyperreactivity [36,38]. Further research on arginase inhibitors that may be suited for endonasal administration may represent a promising area of exploration in the treatment of CRS.

In conclusion, we observed an elevated expression of arginase 2 in sinusal mucosa of patients with CRS, sustaining its possible role in the pathogenesis of this disease, most likely through the decrease of NO via substrate competition with iNOS. We also determined the differentiated expression of ARG2 in the subgroups of patients with CRS defined by Han and detected the highest levels in the non-asthmatic sinusitis without allergy subtype. Its distinct pathology, emphasized by our findings, suggests the need for a more comprehensive classification of CRS that would lead to the development of individualized management strategies and improvement of disease treatment. In this regard, a better understanding of the role of arginase in the pathogenesis of chronic rhinosinusitis will enable the consideration of novel therapeutic strategies. 

## Figures and Tables

**Table 1:** General characteristics of the patients included in the study. AL—allergy, AS—asthma.

	Chronic Rhinosinusitis (*n* = 36)	Control (*n* = 29)	*p*-Value
**Age (years)**	45.8 ± 10.7	42.7 ± 15.4	0.35
**Male gender**	18 (50%)	17 (58.6%)	0.65
**Urban sample**	24 (66.6%)	19 (65.5%)	0.66
**Polyposis**	14 (38.8%)	-	-
**Respiratory allergy**	8 (22.2%)	-	-
Asthma + (AL+ AS+)	3 (8.3%)	-	-
Asthma − (AL+ AS−)	5 (13.8%)	-	-
**Asthma**			
Allergy + (AS+ AL+)	15 (41.6%)	-	-
Allergy − (AS+ AL−)	3 (8.3%)	-	-
Asthma − Allergy −	12 (27.7%)	-	-
(AS − AL−)	16 (44.4%)	-	-
